# Associations between Forced Sexual Initiation, HIV Status, Sexual Risk Behavior, Life Stressors, and Coping Strategies among Adolescents in Nigeria

**DOI:** 10.1371/journal.pone.0155210

**Published:** 2016-05-10

**Authors:** Morenike Oluwatoyin Folayan, Abigail Harrison, Brandon Brown, Morolake Odetoyinbo, Jamila K. Stockman, Ademola J. Ajuwon, Carlos F. Cáceres

**Affiliations:** 1 Institute of Public Health and Department of Child Dental Health, Obafemi Awolowo University, Ile-Ife, Osun State, Nigeria; 2 Department of Behavioral and Social Sciences, School of Public Health, Brown University, Providence, Rhode Island, United States of America; 3 Division of Clinical Sciences, Center for Healthy Communities, University of California Riverside School of Medicine, Riverside, California, United States of America; 4 Positive Action for Treatment Access, Ikeja, Lagos, Nigeria; 5 Division of Global Public Health, Department of Medicine, University of California San Diego, San Diego, California, United States of America; 6 Department of Health Promotion and Education, College of Medicine, University of Ibadan, Ibadan, Oyo State, Nigeria; 7 Center for Interdisciplinary Studies in Sexuality, AIDS and Society, Universidad Peruana Cayetano Heredia, Lima, S.M.P. Lima, Peru; University of Toronto, CANADA

## Abstract

**Objectives:**

Some individuals experience their first sexual intercourse through physically forced sex, which affects the way they experience and cope with stress. We examined differences in sexual risk behavior, experience of stressors, and use of stress-coping strategies among adolescents in Nigeria based on their history of forced sexual initiation and HIV status.

**Methods:**

We analyzed data from 436 sexually active 10–19-year-old adolescents recruited through a population-based survey from 12 Nigerian states. Using Lazarus and Folkman’s conceptual framework of stress and coping, we assessed if adolescents who reported forced sexual initiation were more likely to report HIV sexual risk practices, to report as stressors events related to social expectations, medical care and body images, and loss and grief, and to use more avoidance than adaptive coping strategies to manage stress. We also assessed if HIV status affected experience of stressors and use of coping strategies.

**Results:**

Eighty-one adolescents (18.6%) reported a history of forced sexual initiation; these participants were significantly more likely to report anal sex practices (OR: 5.04; 95% CI: 2.14–11.87), and transactional sex (OR: 2.80; 95% CI: 1.56–4.95). Adolescents with no history of forced sexual initiation were more likely to identify as stressors, life events related to social expectations (OR: 1.03; 95% CI: 0.96–1.11) and loss and grief (OR: 1.34; 95% CI: 0.73–2.65), but not those related to medical care and body images (OR: 0.63; 95% CI: 0.34–1.18). They were also more likely to use adaptive responses (OR: 1.48; 95% CI: 0.62–3.50) than avoidance responses (OR: 0.90; 95% CI: 0.49–1.64) to cope with stress, though these differences were not significant. More adolescents with a history of forced sexual initiation who were HIV positive identified as stressors, life events related to medical care and body images (*p* = 0.03) and loss and grief (*p* = 0.009). Adolescents reporting forced sexual initiation and HIV-negative status were significantly less likely to use religion as a coping strategy (OR: 0.28; 95% CI: 0.09–0.83).

**Conclusion:**

History of forced sexual initiation and HIV status affected perception of events as stressors and use of specific coping strategies. Our study findings could inform best practice interventions and policies to prevent and address forced sexual initiation among adolescents in Nigeria and other countries.

## Introduction

Violence against women and girls is a high-priority global health issue; global estimates suggest that one-third of women have had a lifetime experience of sexual violence [1]. Forced sexual acts include sexually related verbal intimidation, threats of sexual violence, and acts of rape. Sexual activities within traditionally sanctioned forced marriage and those that occur as part of illegal sexual trades such as sexual trafficking and forced prostitution, also fall under this category [1, 2].

More recently, forced sexual initiation, a first sexual intercourse (either vaginal and/or anal) experience resulting from physically forced sex, has been recognized as a distinct sexual violence entity; its prevalence ranges from less than 1% in Japan to nearly 30% in rural Bangladesh [3]. It is reported more frequently among women than men [3] and associated with an increased risk for HIV infection through multiple mechanisms such as deregulation of the protective immune system [4, 5]; sexual risk behaviors such as having sex with multiple or concurrent partners; sex in exchange for money; drug use before or during sex; sex with a high-risk partner [6, 7]; and/or low self-esteem/low competency to refuse unwanted sex and negotiate condom use [8]. In addition, mental health outcomes such as post-traumatic stress disorder and depression, increase the potential for alcohol and/or drug use as a coping mechanism and decrease the ability to negotiate HIV-preventive behaviors [9].

Adolescents and youths are greatly affected by sexual violence [10]. In Nigeria, both male and female adolescents report forced sex, though females are disproportionately affected [11, 12]. In this population, as many as 31.4% female and 5.7% male adolescents who are sexually active report forced sexual initiation [13]. Furthermore, female adolescents living with HIV (ALHIV) are more likely to experience forced sexual initiation than both females not living with HIV and male ALHIV [14–16]. This suggests that HIV infection heightens the social and sexual vulnerability of young girls and adult women, who are already more vulnerable to sexual violence because of gender inequality [17]. Gender inequality results from a complex interplay of factors at the individual, interpersonal, community, and societal levels [3]. Traditional gender and social norms related to male superiority, combined with legal sanctions against violence, are some of the factors that promote perpetration of sexual violence [3].

Those who experience sexual violence in Nigeria are frequently stigmatized rather than offered support and services. In extreme cases, survivors of sexual violence have been killed to alleviate the “shame” associated with rape of a family member [1, 14]. Acquisition of HIV infection among survivors of forced sexual initiation [18] results in double stigma, which takes a toll on the mental health of survivors [13]. Both HIV infection and sexual violence increase the experience of stress and stress-related disorders, and influence survivors’ use of stress-coping strategies [19–22]. Unfortunately, little is known about how adolescent forced sex survivors living in environments that provide limited legal, psychological, or therapeutic support experience life events such as stressors, and how they cope with experienced stress. Even less is known about how adolescent forced sex survivors living with HIV infection cope with stress. Therefore, we chose to investigate how adolescents, including ALHIV, living in an endemically stressful environment like Nigeria [23] perceive and cope with life stressors. Many adolescents in Nigeria live with HIV and its prevalence is increasing in this population [24]. More frequent stressful life events among ALHIV are associated with higher levels of anxiety and depression [25], but ALHIV who adopt adaptive strategies to cope with life stressors show lower levels of anxiety and depression [25].

Our theoretical framework for the study draws on Lazarus and Folkman’s [26] transactional model of stress and coping, which hypothesizes that stress arises when we are unable to cope with life events perceived as threats, and posits that social support is a system that protects people in time of stress. When the source of the threat cannot be managed, individuals often use coping strategies based on emotion or avoidance.

Adolescents depend on trusted, familiar, mature, and friendly social networks for emotional support, especially in times of need [27]. Emotional support encompasses behaviors that contribute to affective well-being, such as listening, expressing love, and showing appreciation. It is therefore unclear how those adolescents who have experienced forced sexual initiation, and who live in environments where stigma associated with rape and HIV infection is high, are able to cope. It is also unclear what coping strategies are employed by adolescents who have experienced forced sexual initiation and who live in communities where the culture of silence and tolerance for rape is very high, and where there are limited and poor quality social networks of support for rape survivors [14].

Coping helps individuals to manage emotional encounters and balance their mental health through cognitive and behavioral responses to challenging life events [28–30]. Individuals could use avoidance coping strategies, which are associated with psychiatric symptoms such as depression; or adaptive coping strategies, which are associated with improved mental health functioning [25, 31, 32]. The use of multiple cognitive and behavioral coping strategies is associated with psychological resilience and self-reported satisfaction with one’s life [33]. The imbalance between a person’s available resources and a given social environment results in stress and reduced coping ability.

We examined differences in sexual risk behavior, life events that cause stress, and the coping strategies adopted by adolescents according to forced sexual initiation status. We also assessed differences in these variables among adolescents who had experienced forced sexual initiation, based on self-reported HIV serostatus.

## Methods

We conducted a secondary analysis of a population-based survey data generated from female and male adolescents aged 10–19 years with diverse ethnic backgrounds in Nigeria. The data were collected in 2012 to determine the sexual and reproductive health needs of ALHIV [34]. Our analysis was limited to 436 (27.7%) sexually active respondents from the 1,574 study participants.

For this study, we examined four potential associations. First, we assessed whether sexual risk behaviors of sexually active adolescents in Nigeria differed based on their experience of forced sexual initiation. Indicators of sexual risk behaviors were engagement in transactional sex, condomless last consensual sexual intercourse, having sex with two or more sexual partners, and having anal sex in the 12 months immediately preceding the survey. Second, we used a 19-item list to test for associations between self-reported forced sexual initiation and adolescents’ perception of common life events as stressors. Third, we used a 13-item list to test for associations between self-reported forced sexual initiation and use of coping strategies to manage stress. Finally, among adolescents reporting forced sexual initiation, we explored if HIV status was associated with identification of any of the 19 common life events as stressors, and usage of any of the 13 stress-coping strategies.

### Recruitment

Details about the sample size, sampling technique and recruitment procedures had been described elsewhere [18]. Briefly, the first stage of recruitment involved the stratification of the 18 states hosting the first 25 antiretroviral therapy treatment sites in Nigeria into six Geopolitical zones. Two states were randomly selected from each of the six geopolitical zones: Lagos, Oyo, Imo, Enugu, Edo, Rivers, Kaduna, Kano, Borno, Adamawa, Plateau, and Benue. Next, two groups of adolescents were recruited from each state. First, ALHIV were recruited through physicians managing people living with HIV at treatment centers (physicians introduced the study to their patients), people living with HIV who attended support groups, and members of the Network of Youths Living with HIV in each state. These contacts shared information about the study and referred ALHIV interested in participating to study team members. Second, adolescents assumed to be HIV negative or whose HIV status was unknown were recruited at youth centers or from those geographical areas where treatment sites were located and which contained large clusters of adolescents. This increased the probability of recruiting both adolescents in- and out-of-school. Recruitment was also conducted in the evenings when the likelihood of seeing large clusters of youth was high. We aimed to recruit 80 adolescents assumed to be HIV negative or with unknown HIV status and those ALHIV from each state.

### Data collection

Data were collected using a face-to-face interviewer-administered structured questionnaire adapted from the 2007 National AIDS and Reproductive Health Survey [35]. The questionnaire was written in English, the official language of the country. Some key words/phrases (including sensitive ones) for each selected community were translated into local dialects during the training of the interviewers. Interviewers used the semi-translated questionnaires as master copies. A similar approach had been used successfully in other studies of adolescents and youth in Nigeria [36–39]. Prior to the fieldwork, the instrument was reviewed by the fieldworkers and by 10 health providers to remove any ambiguity. In addition, the eight field workers went into the field and pilot-tested the instrument to further ensure clarity of statements. Questions were improved for clarity based on the feedback received. To ensure that respondents could interpret the concepts unambiguously, and that the fieldworkers measured the concepts consistently, the instruments included specific notes that explained the concepts.

Information was collected about gender, age, context/reason for first sexual intercourse (“in love,” “having fun,” “peer pressure,” “for money,” and “forced”). All respondents who reported that their first sexual experience was forced were categorized as having had forced sexual initiation. Data were also collected on age at first sexual intercourse, and sexual practices (condom use during last sexual intercourse, sex in exchange for gifts or money, number of sexual partners, and history of anal sexual intercourse) in the 12 months preceding the survey. Study participants were required to confirm whether or not any of the 19 life events had caused them stress and whether or not they had used any of the 13 coping strategies to manage stress.

#### Variables

The independent variables were self-reported forced sexual initiation and self-reported HIV status. The study outcomes were HIV sexual risk practices, perception of life events as stressors, and use of coping strategies. The analytical approach was guided by the transactional theory of stress and coping; we considered how the outcomes of interest (sexual risk behaviors) were influenced by the presence of life stressors and use of coping strategies at the individual level.

To address inconsistencies in responses to questions about sexual activity, decision rules were constructed. Participants who reported any past sexual experience were categorized as “sexually active.” Participants reporting sexual activity before the age of 13 years were defined as having early sexual debut and as possible victims of sexual abuse. This definition of early sexual debut was based on the Nigerian legal system, which defines sexual intercourse with a girl under the age of 13 years as illegal [40].

#### Data handling

Some adolescent girls under 13 years reported sexual activity. We categorized age at sexual initiation as above or below 13 to explore whether victims interpreted underage sex as forced. However, we hypothesized that the effect of forced sexual initiation depends on adolescents’ interpretation of the circumstances surrounding their sexual experiences. Regardless of adolescents’ age at sexual initiation, the relationship between sexual initiation and future life events is likely to be different for those who consider their sexual experiences as coercive compared with those who do not. Consequently, analysis of data on forced sexual initiation was limited to the 81 adolescents who self-reported having had forced sexual initiation, regardless of the respondents’ age.

Similarly, respondents unaware of their HIV status were treated as HIV negative. We assumed that individuals who are aware of their HIV-positive status may respond differently to life events than those who are HIV negative or unaware of their HIV status.

### Data analysis

The basic dimensions underlying the causes of stress and coping strategies for stress were identified using principal components analysis with varimax rotation. Items that loaded at 0.40 or greater on only one factor were retained. Based on the factor analysis, new variables relating to each factor were formed by summing the values of the original variables with the highest loadings on that factor. The sum of the variables was then standardized by dividing it by the number of included variables. Cronbach’s alpha, which measures a scale’s validity, was also calculated for each scale.

#### Factorial validity for stress-causing items

Three factors were identified. Factor I, categorized as “Social expectations,” contained five of the 19 items, and accounted for 51% of the variance. Factor II, categorized as “Medical care and body image,” contained five of the 19 items, and accounted for 35% of the variance. Factor III, categorized as “Loss and grief,” contained three of the 19 factors and accounted for 28% of the variance. The other six items loaded highly on more than one factor; therefore, they were assigned to Factors I, II, and III based on were divided into the three groups based on consensus among the statisticians and researchers.

#### Factorial validity for stress-coping items

Two factors were identified. Factor I, categorized as “Adaptive response,” contained six of the 13 items and accounted for 64% of the variance. Factor II, categorized as “Avoidance responses,” contained three of the 13 items and accounted for 51% of the variance. The other four items loaded highly on more than one factor; therefore, they were assigned to Factors I and II based on consensus among statisticians and researchers.

#### Bivariate and inferential analysis

Pearson’s chi-square test and Fisher’s exact test (when a cell was less than 5) were used to test the significance of associations between variables. Logistic regression analysis was used to determine the odds (for adolescents who reported forced sexual initiation and those who did not) of (a) engaging in HIV-risk sexual practices, (b) identifying a life event as a stressor, and (c) using specific stress-coping strategies. Regression analysis was also used to estimate (for participants reporting forced sexual initiation who were HIV positive and those who were not) (d) identified stressful life events, and (e) stress-coping strategies. All “no” responses were excluded from the tests of associations. Statistical significance was defined as *p* ≤ 0.05. Analysis was conducted using STATA version 12.0 (StataCorp LP; College Station, TX).

### Ethical considerations

Ethics approval for the study was obtained from the National Institute of Medical Research Institutional Review Board, the Health Research Ethics Committee of Plateau State, and the Health Research Ethics Committee of the Federal Capital Territory, Abuja. Written informed consent was obtained from adolescents 15 years and older. Written parental consent and participants’ assent were obtained from participants’ aged 10–14 years.

## Results

Among sexually initiated participants, 81 (18.6%) reported forced sexual initiation. Among the remaining 355 participants, reasons for initiation of sex included peer pressure (26.3%), love (25.7%), having fun (21.1%), a financial transaction (3.0%), or marriage (0.2%). Twenty-two respondents (5.0%) gave no reason for sexual initiation.

Table 1 shows the profile of respondents who were sexually active at the time of the survey. Of the 81 respondents who reported forced sexual initiation, 18 (22.2%) had their first sexual intercourse under 13 years of age. Males had significantly lower odds of reporting a history of forced sexual initiation (OR: 0.16; 95% CI: 0.09–0.30). Adolescents who self-reported as HIV positive had significantly increased odds of reporting a history of forced sexual initiation compared with adolescents who self-reported as HIV negative (OR: 1.95; 95% CI: 1.11–3.41).

**Table 1:** Age and HIV status distribution of respondents by history of sexual intercourse.

Variables	Sexually active respondents (N = 436)	Respondents who reported forced sexual initiation (N = 81)
Age of first sexual intercourse: ≤12 years	40 (9.2%)	18 (22.2%)
Age of first sexual intercourse: 13–19 years	330(75.7%)	51 (63.0%)
Do not know age	66 (15.1%)	12 (14.8%)
**Total**	**436 (100%)**	**81 (100%)**
Male	216 (49.5%)	12 (14.8%)
Female	220 (50.5%)	69 (85.2%)
**Total**	**436 (100%)**	**81 (100%)**
HIV positive	170 (39.0%)	31 (38.3%)
HIV negative	71 (16.3%)	10 (12.3%)
Unknown HIV status	152 (34.9%)	18 (22.2%)
Recruited HIV positive but gave no response on HIV status	43 (9.8%)	22 (27.2%)
**Total**	**436 (100%)**	**81 (100%)**

### Forced sexual initiation and sexual risk behavior

Table 2 shows the distribution of study participants by history of forced sexual initiation status and HIV sexual risk practices. Of 81 adolescents who reported a history of forced sexual initiation, 25 (30.9%) had engaged in transactional sex, 44 (53.3%) had not used a condom at the last sexual intercourse act, 15 (18.5%) had more than one sexual partner, and 11 (13.6%) had had anal sex in the last 12 months. Adolescents with a history of forced sexual initiation were significantly more likely to engage in transactional sex (OR: 2.80; 95% CI: 1.56–4.95) and anal sex in the 12 months preceding the study (OR: 5.04; 95% CI: 2.14–11.87) compared with adolescents who had no history of forced sexual initiation. Additionally, a higher proportion of sexually active females who reported forced sexual initiation had had more than one sexual partner in the last 12 months compared with sexually active females who did not report forced sexual initiation (41.6% vs. 37.9%; *p* = 0.001).

**Table 2:** History of forced sexual initiation and HIV-risk sexual behavior.

Variables	Sexually active adolescents who reported a forced sexual initiation (N = 81)	Sexually active adolescents who did not report forced sexual initiation (N = 355)	Total number (N = 436)	P value
Transactional sex	25 (30.9%)	64 (18.0%)	89 (20.4%)	0.008
Condom used at last sexual intercourse act	37 (46.7%)	127 (35.8%)	164 (37.6%)	0.10
Multiple sexual partners	15 (18.5%)	72 (20.3%)	87 (20.0%)	0.72
Anal sex in the last 12 months	11 (13.6%)	14 (3.9%)	25 (5.0%)	0.001

### Forced sexual initiation and perception of life events as stressful

Overall, the proportion of adolescents with a history of forced sexual initiation did not significantly differ from the proportion of adolescents without such a history who identified life events associated with social expectations (*p* = 0.25), medical care and body images (*p* = 0.19), and loss and grief (*p* = 0.11) as stressors.

Table 3 shows the distribution of study participants by history of forced sexual initiation status and the 19 stress factors examined. Significant differences were observed only for two life stressors: when compared with sexually active adolescents who had reported forced sexual initiation, sexually active adolescents who had no history of forced sexual initiation were more likely to identify not having friends as a stressor (OR: 3.63; 95% CI: 1.51–8.71). Similarly, when compared with sexually active adolescents who had no history of forced sexual initiation, sexually active adolescents who had reported forced sexual initiation were more likely to identify someone saying something about them that they did not like as a stressor (OR: 2.18; 95% CI: 1.29–3.66).

**Table 3:** Experience of life stressors, forced sexual initiation, and self-reported HIV status.

Life Events as Stressors	History of forced sexual initiation among sexually active adolescents (N = 436)				HIV status of sexually active adolescents with a history of forced sexual initiation (N = 81)			
	History of FSI (n = 81)	No History of FSI (n = 355)	X^2^	P value	HIV positive (n = 53)	HIV negative (n = 28)	X^2^	P value
SOCIAL EXPECTATIONS								
Pressures from peers to do what I do not believe in	30 (37.0%)	147 (41.1%)	0.52	0.47	16 (30.2%)	14 (50.0%)	3.08	0.08
Pressure from parents to do what I do not believe in	21 (25.9%)	96 (27.0%)	0.04	0.84	13 (24.5%)	8 (28.6%)	0.16	0.69
Need to excel in my academic performance in school	15 (18.5%)	89 (25.1%)	1.56	0.21	8 (15.1%)	7 (25.0%)	1.19	0.28
Conforming to religious beliefs	8 (9.9%)	53 (14.9%)	1.40	0.24	6 (11.3%)	2 (7.1%)	0.36	0.71
Extra work from school or home	20 (24.7%)	96 (27.0%)	0.19	0.67	14 (26.4%)	6 (21.4%)	0.25	0.62
Argument with a friend or family member	14(17.3%)	81 (22.8%)	1.18	0.28	9 (17.0%)	5 (17.9%)	0.010	0.92
Someone saying something about me that I don’t like	34 (42.0%)	93 (26.2%)	7.95	**0.005**	25 (47.2%)	9 (32.1%)	1.70	0.19
**Overall**	62 (76.5%)	249 (70.1%)	1.32	0.25	38 (71.7%)	24 (85.7%)	2.00	0.16
MEDICAL CARE AND BODY IMAGE								
Having to visit the hospital regularly	31 (38.3%)	102 (28.7%)	2.83	0.09	24 (45.3%)	7 (25.0%)	3.19	0.07
Having to take drugs regularly	29 (35.8%)	122 (34.4%)	0.06	0.81	22 (41.5%)	7 (25.0%)	2.17	0.14
Dealing with stigma and discrimination	32 (39.5%)	113 (31.8%)	1.75	0.19	25 (47.2%)	7 (25.0%)	3.77	0.052
Body looks different from others	15 (18.5%)	51 (14.4%)	0.89	0.35	8 (15.1%)	7 (25.0%)	1.19	0.28
Not adhering to my medications	13 (16.0%)	59 (16.6%)	0.02	0.90	9 (17.0%)	4 (14.3%	0.10	1.00
**Overall**	43 (53.1%)	160 (45.1%)	1.70	0.19	36 (67.9%)	7 (25.0%)	4.74	**0.03**
LOSS AND GRIEF								
Thinking about death	26 (32.1%)	83 (23.4%)	2.67	0.10	21 (39.6%)	5 (17.9%)	3.98	**0.05**
Handling the death of a loved one	20 (24.7%)	63 (17.7%)	2.06	0.15	13 (24.5%)	7 (25.0%)	0.002	0.96
May never have children	10 (12.3%)	31(8.7%)	1.01	0.32	6 (11.3%)	4 (14.3%)	0.15	0.73
Do not have friends	10 (12.3%)	227 (63.9%)	70.77	**<0.01**	8 (15.1%)	2 (7.1%)	1.07	0.48
Not in a sexual relationship with someone	5 (6.2%)	26 (7.3%)	0.13	0.72	2 (3.8%)	3 (10.7%)	1.52	0.33
Do not have good relationship with my care provider	6 (7.4%)	30 (8.5%)	0.09	0.76	6 (11.3%)	0 (0.0%)	3.42	0.08
Having problems with boy/girlfriend or spouse	23(28.4%)	117 (33.0%)	0.63	0.43	15 (28.3%)	8 (28.6%)	0.0007	0.98
**Overall**	53 (65.4%)	198 (55.8%)	2.52	0.11	40 (75.5%)	13 (46.4%)	6.83	**0.009**

### HIV status and causes of stress in adolescents with a history of forced sexual initiation

Overall, 62 (76.5%), 43 (53.1%), and 53 (65.4%) of the 81 adolescents with a history of forced sexual initiation identified as stressors common life events related to social expectations, medical care and body image, and loss and grief, respectively. The presence of HIV infection increased the likelihood of identifying those life events related to medical care and body images (*p* = 0.03) and loss and grief (*p* = 0.009) as stressors. Table 3 shows the distribution of study participants by history of forced sexual initiation status, self-reported HIV status, and the 19 stress factors. Among adolescents who reported forced sexual initiation, those reporting HIV-positive status were more likely than those without HIV infection to identify thinking about death as a stressor (*p* = 0.05).

### Forced sexual initiation and coping strategies

Table 4 shows the distribution of study participants by history of forced sexual initiation status and the 13 stress-coping strategies. There was no significant difference in the proportion of adolescents with or without a history of forced sexual initiation who used adaptive (*p* = 0.52) and avoidance (*p* = 0.81) coping strategies. The top three coping strategies used by adolescents were seeking social support (55.3%), religion (51.2%), and acceptance (38.2%). The least commonly used strategy was alcohol/drug use (9.3%). There were no significant differences observed in the proportion of adolescents with or without a history of forced sexual initiation who used any of the 13 coping strategies.

**Table 4:** Reported use of coping strategies by history of forced sexual initiation and self-reported HIV status.

Coping strategies	History of forced sexual initiation among sexually active adolescents (N = 436)				HIV status of sexually active adolescents with a history of forced sexual initiation (N = 81)			
	History of FSI (n = 81)	No History of FSI (n = 355)	X^2^	P value	HIV positive (n = 53)	HIV negative (n = 28)	X^2^	P value
ADAPTIVE RESPONSE								
Seek social support	42 (52.5%)	181 (51.0%)	0.02	0.89	27 (50.9%)	15 (53.6%)	0.05	0.82
Planful problem solving	44 (55.0%)	157 (44.2%)	2.71	0.10	27 (50.9%)	17 (60.7%)	0.70	0.40
Religion	46 (57.5%)	166 (46.8%)	2.66	0.10	36 (68.0%)	10 (35.7%)	7.75	**0.005**
Positive appraisal	29 (36.3%)	111 (31.3%)	0.62	0.43	18 (34.0%)	11 (39.3%)	0.23	0.64
Acceptance	34 (42.5%)	145 (40.8%)	0.03	0.85	21 (39.6%)	13 (46.4%)	0.35	0.56
Humor	19 (23.8%)	91 (25.6%)	0.17	0.68	12 (22.6%)	6 (21.4%)	0.02	0.90
Journaling	4 (5.0%)	39 (11.0%)	2.71	0.10	3 (5.7%)	1 (3.6%)	0.17	1.00
**Overall**	68 (84.0%)	287 (80.8%)	0.42	0.52	45 (84.9%)	23 (82.1%)	0.001	0.97
AVOIDANCE RESPONSES								
Confrontation	35 (43.8%)	152 (42.8%)	0.004	0.95	23 (43.4%)	12 (42.9%)	0.002	0.96
Escape/denial	14 (17.5%)	73 (20.6%)	0.44	0.51	9 (17.0%)	5 (17.9%)	0.010	0.92
Mental disengagement	25 (31.3%)	91 (25.6%)	0.92	0.34	17 (32.1%)	8 (28.6%)	0.11	0.75
Behavioral disengagement	23 (28.8%)	101 (28.5%)	0.0001	0.99	15 (28.3%)	8 (28.6%)	0.0007	0.98
Alcohol/drug use	14 (17.5%)	57 (16.1%)	0.07	0.79	8 (15.1%)	6 (21.4%)	0.51	0.47
Suppression of competing activities	16 (20.0%)	54 (15.2%)	1.01	0.32	9 (17.0%)	6 (21.4%)	0.24	0.62
**Overall**	52 (64.2%)	233 (65.6%)	0.06	0.81	38 (71.7%)	14 (50.0%)	3.75	0.053

### HIV status and coping strategies in adolescents reporting forced sexual initiation

Table 4 also shows the distribution of study participants by history of forced sexual initiation status, self-reported HIV status, and stress-coping strategies. Overall, 68 (84.0%) and 52 (64.2%) of the 81 adolescents with a history of forced sexual initiation identified adaptive and avoidance stress-coping strategies respectively used. Although there was no significant difference in the proportion of adolescents with and without HIV infection who used adaptive strategies (*p* = 0.97), there was a trend for more HIV-positive adolescents to use avoidance strategies (*p* = 0.053). In terms of specific strategies, adolescents with HIV-negative status were significantly less likely to use religion as a coping strategy (OR: 0.28; 95% CI: 0.09–0.83).

### Association between forced sexual initiation stressors and use of coping strategies

Table 5 highlights the outcomes of the multivariate logistic regression analysis to determine predictors of use of coping strategies and identifiers of stressors among adolescents who reported a history of forced sexual initiation, adjusting for HIV status. No significant associations were found, but certain trends became apparent. For example, adolescents who had no history of forced sexual initiation seemed less likely to identify aspects of medical care and body image as stressors (AOR): 0.63; 95% CI: 0.34–1.18) or to use avoidance responses (AOR: 0.90; 95% CI: 0.49–1.64) as coping strategies compared with adolescents who had a history of forced sexual initiation.

**Table 5:** Logistic regression model determining stressors and coping strategies used by adolescents with a history of forced sexual initiation.

Variables	History of forced sex initiation	Adjusted Odds Ratio	95% CI	P values
**Life stressors**				
Social expectations	Yes (76.5%)	1	**-**	**-**
	No (33.5%)	1.03	0.96–1.11	0.39
Medical care and body image	Yes (53.1%)	**1**	**-**	**-**
	No (46.9%)	0.63	0.34–1.18	0.15
Loss and grief	Yes (65.4%)	1	**-**	**-**
	No (34.6%)	1.39	0.73–2.65	0.32
Coping strategies				
Adaptive responses	Yes (84.0%)	1	-	-
	No (16.0%)	1.48	0.62–3.50	0.38
Avoidance responses	Yes (64.2%)	1	-	-
	No (35.8%)	0.90	0.49–1.64	0.72

## Discussion

Our study explored the correlates of forced sexual initiation in relation to HIV risk, identification of life events as stressors, and coping mechanisms among adolescents in Nigeria; it also explored the ways in which HIV infection may modify these relationships.

First, we found differences in some but not all HIV sexual risk behaviors when comparing adolescents who had a history of forced sexual initiation with those who had no such history. In contrast to previous studies [41, 42], we did not observe a difference in condom use in the last sexual act between adolescents who reported forced sexual initiation and those who did not. However, the general low condom use in Nigeria, particularly in youths [43], makes it unlikely that meaningful changes could be observed.

Similarly, although we may be among the first to report forced sexual initiation in association with anal sex in adolescents, a prior report from India has identified an association between sexual violence and anal sex in adult female sex workers [44]. New evidence of a direct association between anal sex and forced sex is emerging [8], and the concept of forced sexual initiation as a distinct form of sexual violence is a recent one. The lack of previous reports of such a relationship may simply indicate that other studies have not explored this issue. In fact, qualitative evidence shows that sexual violence and coercion are a common correlate of anal intercourse [13, 45, 46] and pose increased risk for HIV given the risk of the perpetrator being HIV-infected [47], the increased risk of genital injuries among adolescents [48], and possibly a stress-mediated increase in HIV risk among survivors [8, 49].

In addition, adolescents who had experienced forced sexual initiation were more likely to engage in transactional sex. Although prior studies have identified an association between sexual violence and transactional sex [50–54], with transactional sex increasing the risk of sexual violence because of differences in the power dynamics of the actors involved [8], this seems to be the first report of an association between a history of forced sexual initiation and transactional sex. In accord with Stockman et al. [9], we hypothesized that individuals with a history of forced sexual initiation engage in transactional sex because of low self-esteem and vulnerability to depression [55]. Alternatively, both forced sexual initiation and engagement in transactional sex may reflect contexts of structural vulnerability that these adolescents have little opportunity to modify without better policies around sexuality, gender, and HIV [56]. Such policies should integrate HIV responses and efforts to promote gender equity, as well as sexual and reproductive health and rights, as an integrated response that enhances the independent access of adolescents to quality prevention, treatment, and care services.

Also, our explanation for these study findings focuses on mechanisms of social support or the lack thereof. Adolescents who reported forced sexual initiation were less likely to be concerned about having friends and more likely to be concerned about what others say about them. Their lesser concern about friendship may reflect an increased tendency for isolation or a general lack of social support. As part of a culture in which body image is important for young people [57], these youths are more likely to report issues in this domain (e.g., body looks different from others, problems with boy/girlfriend or spouse). Their heightened concern about what others say about them may increase their tendency for isolation to prevent further pain.

Furthermore, our findings suggest that among adolescents who report forced sexual initiation, the added experience of HIV infection leads them to identify more life events as stressful and adopt more avoidance coping strategies [58, 59]. The high level of HIV-related stigma in a country like Nigeria [60] may increase the likelihood of psychosocial distress, which may be further compounded by HIV infection [61]. These findings largely support the theories of stress and coping that guided this analysis, and emphasize the complex relationships between personal control, stress, and coping. Also, these findings underscore the importance of connecting ALHIV who experience forced sexual initiation with care and prevention programs [9, 62].

Next, in contrast to the findings of Bal et al. [63], we found that the coping strategies used by adolescents who reported forced sexual initiation did not differ significantly from those used by adolescents who did not report forced sexual initiation. We did not observe the increase in the use of avoidance coping strategies that was reported by Bal et al. [64] for adolescents from Belgium. The observed difference in the reports of the two studies may reflect culturally specific differences, a finding supported by situational models of stress and coping [33]. In Nigeria, individuals who are raped are often labeled negatively [14]. Therefore, rape survivors may choose not to change their visible behavior, but adopt instead, mental and emotional strategies that involve denial and avoidance to cope with stress. Our observation that adolescents who reported forced sexual initiation had reduced interest in having friends highlights the use of avoidance coping strategies to cope with stress and corroborates our hypothesis. The limited availability of social and community support structures for young people who face sexual violence could also explain adolescents’ choice of coping strategies.

Additionally, we found that a history of forced sexual initiation and HIV-positive status increased adolescents’ concerns about death and increased the tendency to use religion to cope with stress. Seeking solace in religion when faced with stressful life events may be associated with concerns about death, frequent hospital visits, and relatively poor state of health. Death and concerns about poor health status are constant threats for ALHIV in sub-Saharan Africa, many of many of whom are often lost during their transition from child to adult care [65].

Finally, our findings clearly suggest that adolescents who report forced sexual initiation need social support, as they are likely to have poorer physical [66] and psychological [67] health. This need is even greater for adolescents who report both a history of forced sexual initiation and HIV-positive status. Although the buffering hypothesis postulated by Cohen and Wills [68] proposes that social support probably protects people in times of stress, Murphy et al. [25] found this not to be true for ALHIV in the United States. Murphy et al. [25] noted that adolescents receive their primary social support from friends and families, who may not relieve anxiety but exacerbate it. Further, as suggested by the transactional model used in our study, stress and coping may manifest differently according to the situation.

The findings of this study have public health implications for Nigeria, where the prevalence of sexual violence [13] and HIV [69] are high, yet public discussion about sexual violence and its preventive and palliative measures including the use of HIV post-exposure prophylaxis [70], is insufficient [13]. The association between a history of forced sexual initiation and high-risk sexual behavior in this study population increases the risk of sexually transmitted infections/HIV and unwanted pregnancy. In addition, poorly managed mental health disorders resulting from forced sexual initiation increase the risk that affected adolescents will develop into maladjusted adults [71].

This study had a number of limitations. First, it was based on secondary data analysis so the power of the study to make inferential analysis was limited. Second, there was only a small sample of adolescents who reported forced sexual initiation, thereby increasing the chances of type I statistical error. The sample size for the analysis on forced sexual initiation was also small (27% of the population recruited for the study), which undermines its power to detect differences in stressors or coping strategies used between the comparative groups. Third, as stigma is associated with both HIV infection and forced sex, standard recruitment procedures were not applicable. Thus, the recruitment strategies for ALHIV and adolescents who were HIV negative were suboptimal. Also, our study did not evaluate the impact of stress and coping strategies used by adolescents who experienced forced sexual initiation and who may be HIV positive or negative based on their physical and psychological health. Additionally, the study relied on self-reported HIV status of the study participants recruited as HIV negative. Finally, data were collected using a web-based instrument that was not validated for use in the study setting. To address this, we conducted a factorial analysis to validate the content of the instrument.

Despite these limitations, this study provides important insights into the risks and stress experienced by adolescents in Nigeria who have had forced sexual initiation. It also provides insight into how the HIV status of those who reported forced sexual initiation influences the experience of stress and choice of stress-coping strategies. The findings generated by this study provide exploratory evidence of the association between a history of forced sexual initiation, higher sexual risk-taking behavior, more frequent experience of stress, and specific coping patterns.

Importantly, the hypothetical social psychological pathways by which forced sexual initiation might affect the lives of people through emotional pain, stress, and low self-esteem may function concomitantly with structural pathways by which, for example, social vulnerability increases the likelihood of both forced sexual initiation and higher sexual risk [3]. Structural factors such as poverty, lack of social support, lack of family support owing to parental disease or death, or lower access to services, may favor higher sexual vulnerability that is expressed in increased sexual violence and sexual risk. Future studies should explore the roles of both types of mechanisms, the synergies between them, and how these affect young people in Nigeria [56, 72]. A better understanding of these complex social problems should guide policy changes so that high social vulnerability contexts are rapidly identified and specific protective measures are established early to reduce the likelihood of forced sexual initiation among adolescents. Also the policy changes can facilitate access of survivors of forced sexual initiation to comprehensive services that helps reduce sexual risk and prevent harmful effects on their mental health.
